#  Frequency, Causes, and Findings of Brain CT Scans of Neonatal Seizure at Besat Hospital, Hamadan, Iran

**Published:** 2015

**Authors:** Fateme EGHBALIAN, Bahman RASULI, Farnaz MONSEF

**Affiliations:** 1 Pediatric Department, Hamadan University of Medical Sciences, Besat Hospital. Hamadan, Iran; 2 Hamadan University of Medical Sciences, Besat Hospital. Hamadan, Iran

**Keywords:** Neonatal seizure, Hypoxic- ischemic encephalopathy, Brain CT scan

## Abstract

**Objective:**

Neonatal seizures are the most common neurological symptoms and often signal an underlying serious neurologic condition. This study determines the frequency of neonatal seizure, predisposing factors, and brain computed tomography (CT) scan findings.

**Materials & Methods:**

In a descriptive cross-sectional study, we evaluated all neonates with seizures who had been hospitalized in Besat hospital from 2007–2012. All data were gathered with questionnaires and used to compare with statistical tests by SPSS (ver 16).

**Results:**

141 (4.08%) neonates (M:F; 1:2.2) were diagnosed with neonatal seizures. From the total number of 3,452 neonatal hospitalization, 78% of neonates with seizures were less than 10 days old and 60.3% of infants were born from natural vaginal delivery. As the most common cause, hypoxic-ischemic encephalopathy in this study was associated with 31.3% (n=44) of neonatal seizures and with the highest mortality rate (n=6). Among admitted neonates with seizures, the overall mortality rate was 12.8% (18 cases). A total of 33.3% of patients (47 cases) had abnormal CT scan reports and 24.8% (35 cases) of patients were not evaluated with a CT scan. Hypoxic-ischemic encephalopathy (47%) and local ischemic changes (25.5%) were the most common findings in the CT scans of neonates with seizures.

**Conclusion:**

There was a significant correlation between neonatal seizures and delivery circumstances (p-value < 0.05). Therefore, with improvement of obstetric and delivery circumstances, early detection of predisposing factors and other rare conditions, and rapid effective treatment of these contributing factors, the rate of neonatal seizure in this period can be reduced.

## Introduction

Seizures are one of the first symptoms for neurological dysfunction in infants. Neonatal seizures underlie conditions that could be manageable from a prompt diagnosis and treatment; whereas, any misdiagnosis could lead to irreversible damage to neurological system. Neonatal seizures correlate with neonatal death and survivors could suffer from neurological sequelae, epilepsy, and growth disorders in the future (1). Previous studies reported 1.5–5.5 neonatal seizures per 1,000 live births. Differences between descriptions of seizure, misdiagnosis, and studies in different populations may cause these statistical differences (2-4). Clinically, neonatal seizures occur because of repetitious and abnormal changes in brain function and usually occur in the first 28 days of term infant or until week 44 of reclaimed age of preterm infants (5). 

Studies on electroencephalograms (EEGs) explain some clinical symptoms in infants without any changes in EEG patterns, i.e., silent seizures have no significant correlation with EEG whereas generalized seizures (tonic, clonic, myoclonic) have EEG abnormal findings. However, the value of these findings in neonatal seizure evaluation remain unclear (6). Incidences of seizures correlate with preterm labor, low-birth weight, and severity of underlying diseases (2-7). 

Hypoxemic-ischemic encephalopathy is the most common cause (about 2/3) of seizures in newborns (2-4, 8). Metabolic disorders, brain hemorrhages, and infections (sepsis, meningitis) are the other causes of neonatal seizures. Recent studies on animal samples have demonstrated that short and repetitious seizures in infants could cause resistant brain damage, cognitive disorders, behavioral changes, anxiety, and increased risk of epilepsy (9-17). Laboratory and radiological studies should be done based on suspected differential diagnosis to prevent neurological complications. Blood sugar, blood, and cerebrospinal fluid (CSF) cultures should be evaluated to rule-out meningitis. Additionally, serum levels of Na, K, P, and Mg should be analyzed (18-21). The other laboratory studies and radiological modalities should be performed based on certain patient symptoms and situations. CT scans and MRIs could demonstrate particular brain lesions that have the potential to cause seizures (19, 22- 24). 

Neonatal brain ultrasounds could be used to screen for gross brain lesions but they are not specific enough and often require additional imaging studies. Although performing an MRI is difficult for infants, it is preferred over CT scans because of its non-ionizing magnetic waves (25). CT scans are used for appropriate diagnosis of acute causes of seizures whereas the MRI is resistant to treatment and repetitious seizures (26). This study determines the etiology of neonatal seizures and studies the specific role of brain CT scans abnormal findings in evaluation and treatment of seizure. 

## Materials & Methods

In this cross-sectional descriptive study of high-risk infants with seizures admitted to the Neonatal Intensive Care Unit (NICU) of Besat faculty hospital in Hamadan from first of July 2006 until 31 December 2012 were included in this study. An informed written consent from the parents of the neonates was prepared and the aim of this study was explained to them. The inclusion criterion was suspected seizure on observation of symptoms by a doctor. Neonatal seizures were diagnosed clinically by a neonatologist and categorized as generalized (tonic, clonic) seizures, multifocal, partial seizures and jerking movements comprising abnormalities of gaze or extra ocular movements that are insensitive to outside stimulation. Sleep-related muscular activities or jitteriness and other non-seizure gestures were distinguished from neonatal seizures based on the absence of orbital and ocular movements and normal sensorium as well as a lack of autonomic changes and cessation of movements on holding the affected limbs and documented by lack of EEG changes. 

Information for each patient was recorded in specific forms comprising different sections that included the following: 1. demographic information (gender, age, weight of neonates, antenatal history of intrauterine infections, maternal drug intake during pregnancy, delivery details (normal vaginal or C-section and complications), and history of resuscitation); 2. type of seizure (Generalized, Multifocal, Partial, Subtle); 3.causes of seizure (hypoxemic-ischemic encephalopathy (HIE), intraventricular hemorrhage (IVH), subarachnoid hemorrhage (SAH), meningitis, sepsis, metabolic disorders, brain anatomical malformations); 4. short-term outcome of neonate seizure during 30 days follow up after occurrence of seizures (discharge or expire); and 5. CT scan findings, if performed, according to a certified radiologist report (Brain hemorrhage, local ischemic lesions in brain tissue, hypoxemic-ischemic encephalopathy, and brain anatomical malformation). 

First-line assessments including serum calcium, blood glucose, serum sodium, serum magnesium, complete blood count, and cerebrospinal fluid (CSF) analysis for any evidence of infection, liver function tests (LFTs), Serum urea/ creatinine, cranial ultrasound (for edema, intraventricular hemorrhage, hydrocephalus, or evidence of gross brain malformation), an EEG was performed in all neonates. Second-line assessments including blood culture, CT scan (done in patients with bulging fontanelles, focal neurological deficit, and resistant seizures to anticonvulsant drugs), TORCH antibody titer, Coomb’s test, reticulocyte count, and urine analysis were done in selected patients selected by history, physical examination, and initial assessments to reach the final diagnosis. Hypoglycaemia was defined as a blood glucose level less than 40 mg/dl, hypocalcaemia as serum calcium level less than 7.5 mg/dl. 

Statistical analysis was performed following sample selection, collecting initial data, and the variables were analyzed using descriptive and presumption methods such as mean, Chi-square, Crus Calvarias, and Man U Whitney tests with SPSS (ver 16). A P < 0.05 was considered significant. 

## Results

In this study, 141 infants who suffered from seizures were selected from 3,452 infants who were hospitalized in the NICU for different reasons. A total of 97 patients were female and the remaining 44 patients were male (M:F; 1:2.2). The average age of infants was 6.91 ± 6.29 days old; while 110 infants (78%) were less than 10 days old. There were only 34 infants (24%) who were less than 2500 gr. A total of 85 infants (60.3%) were born with normal vaginal delivery and 56 infants (39.7%) with C-Section. A total of 66 infants (46.8%) were born following a difficult delivery (Table1). 

The most common seizure was generalized and multifocal seizure with 70 cases (49.7%); while 56 infants (39.7%) suffered subtle seizure; and partial seizures happened in 15 infants (10.6%) (Table2). Generalized or multifocal seizures comprised tonic and then colonic seizures. Hypoxic-ischemic encephalopathy was the major causal factor of seizures with 44 cases (31.3%), 31 cases (22%) had metabolic disorders including hypoglycemia or hypocalcaemia, 16 cases (11.4%) had infectious underlying factor, 11 cases (7.8%) had brain hemorrhage, 2 cases were diagnosed (1.4%) with brain structural abnormalities, and one case (0.7%) was due to the mother’s drug abuse and the remaining 36 cases (25.5%) had unknown reasons for the seizures (Table 2). It should be mentioned that from the whole of 16 cases in which infection was the causal factor of neonatal seizure, one was diagnosed with meningitis and the other 15 were diagnosed with sepsis (8 cases of positive blood culture and 7 cases of positive urine culture). Among the 11 neonates with the diagnosis of intracranial hemorrhage, intraventricular hemorrhage was the causal factor in 9, and subarachnoid hemorrhage in the remaining 2 cases. In 31 cases with metabolic disorders as the cause of seizure, hypoglycemia was a causal factor in 18 cases, and the remaining 13 cases were caused by hypocalcemia. A total of 18 (12.8%) neonates with seizure died during hospitalization and hypoxic-ischemic encephalopathy was associated with the highest mortality rate (P-value < 0.05) (Table 3). 

**Fig 1:**
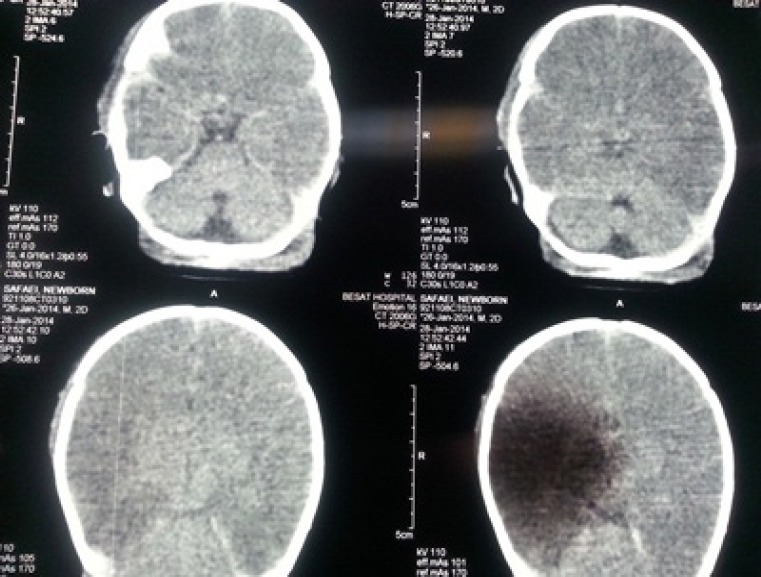
Unenhanced CT, performed on day 2, demonstrates a hypo dense area in the middle part of the right middle cerebral artery territory in the right hemisphere. The right parietal soft tissue swelling was seen. Gray-white matter differentiation was lost diffusely in favor of hypoxemic-ischemic injury.

**Fig 2:**
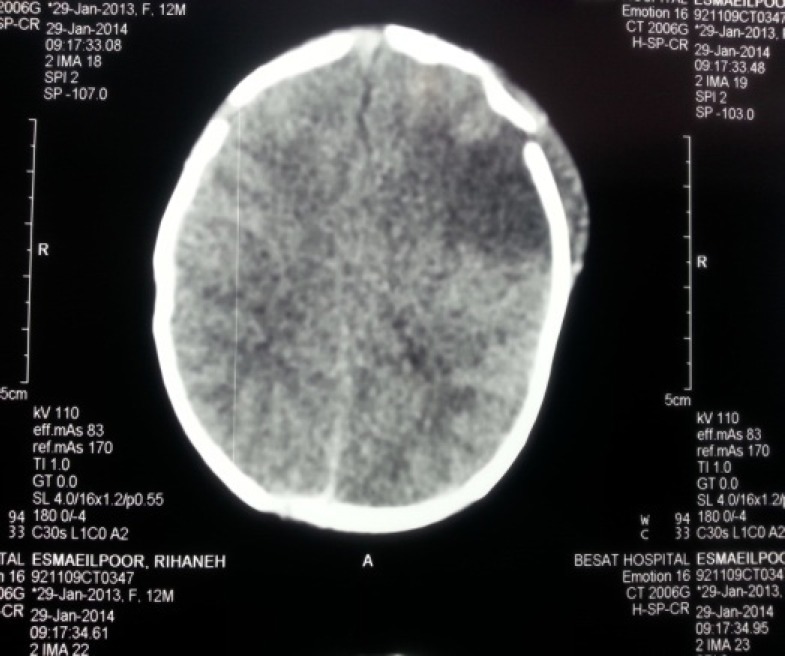
Unenhanced CT, performed on day 6, demonstrates a hypodense area in the anterior part of the left middle cerebral artery territory in the left frontoparietal region related to local ischemic changes. Adjacent cephalhematoma was also present.

In the present study, a positive and significant correlation was seen between the etiology of neonatal seizures and infant age at the time of seizure, delivery circumstances, and early complications (P-value < 0.05). Additionally among different neonatal variables, there was a positive and significant correlation between the low birth weight and the early complications that appeared following seizures (P-value < 0.05). A total of 47 cases (33.3%) had abnormal CT scan results and CT scans were not performed in 35 cases (24.8%). Brain CT scan results according to radiologist were as follows: hypoxemic-ischemic encephalopathy in 22 cases (47%) (Fig. 1); local ischemic lesions in 12 cases (25.5%) (Fig 2); brain hemorrhage in 11 cases (23.3%); and brain structural malformations (one case had holoprosencephaly and the other had hydranencephaly) in 2 cases (4.2%). A positive and significant correlation was seen between the type of lesion that was found in Brain CT scans and the causal factor of neonatal seizures (P-value < 0.05). 

As we can see, there is a significant correlation between difficult deliveries and the etiology of seizures (P < 0.05). 

There is no significant correlation between the type and the etiology of seizures (P > 0.05). 

There is a significant correlation between the outcome and the etiology of neonatal seizures (P < 0.05).

**Table 1:** Correlation between Delivery History and Etiology of Neonatal Seizures, Neonatal Intensive Care Unit of Besat Hospital, Hamadan

**Total**	**Delivery type**		**Delivery condition**		** Delivery history**
	**C-section**	**NVD**	**Without difficulty**	**Difficult delivery**	**Seizure etiology**
44 (31.3%)	22 (50%)	22 (50%)	15 (34.1%)	29 (65.9%)	Hypoxemic-ischemic encephalopathy
31 (22%)	10 (32.3%)	21 (67.7%)	18 (58.1%)	13 (41.9%)	metabolic
16 (11.3%)	10 (62.5%)	6 (37.5%)	12 (75%)	4 (25%)	infection
11 (7.8%)	2 (18.2%)	9 (81.8%)	8 (72.7%)	3 (27.3%)	CNS hemorrhage
2 (1.4%)	0 (0.0%)	2 (100%)	2 (100%)	0 (0.0%)	Brain structural malformation
1 (0.7%)	0 (0.0%)	1 (100%)	1 (100%)	0 (0.0%)	Maternal drug use
36 (25.5%)	12 (33.4%)	24 (66.6%)	19 (52.8%)	17 (47.2%)	unknown
141 (100%)	56 (39.7%)	85 (60.3%)	75 (53.2%)	66 (46.8%)	Total
	0.155		0.041		P-value

**Table 2:** Correlation between Seizure Type and Etiology, Neonatal Intensive Care Unit of Besat Hospital, Hamadan

**Total**	**Unknown**	**Maternal drug use**	**Brain structural malformation**	**CNS Hemorrhage**	**Infection**	**Metabolic**	**Hypoxemic-ischemic encephalopathy**	** Seizure etiology**
								**Seizure kind**
70 49.7%	17 47.2%	1 100%	1 50.0%	7 63.7%	8 50.0%	15 48.4%	21 47.7%	Generalized & Multifocal
15 10.6%	3 8.3%	0 0.0%	0 11.1%	3 27.3%	0	2 6.4%	7 15.9%	Partial
56 39.7%	16 44.5%	0 0.0%	1 50.0%	1 9.0%	8 50.0%	14 45.2%	16 36.4%	Subtle
141 100%	36 25.5%	1 0.7%	2 1.4%	11 7.8%	16 11.3%	31 22%	44 31.3%	Total
		0.455						P-Value

**Table 3:** Correlation between The Outcome and The Etiology of Seizures, Neonatal Intensive Care Unit of Besat Hospital, Hamadan

**Total**	**Unknown**	**Maternal drug use**	**Brain structural malformation**	**CNS Hemorrhage**	**Infection**	**Metabolic**	**Hypoxemic-ischemic encephalopathy**	** Seizure etiology**
								**outcome**
123 87.2%	32 88.8%	1 100%	0 0.0%	7 63.7%	15 93.7%	30 96.7%	38 86.3%	discharged
18 12.8%	4 11.2%	0 0.0%	2 100%	4 36.3%	1 6.3%	1 3.3%	6 13.7%	expired
141 100%	36 25.5%	1 0.7%	2 1.4%	11 7.8%	16 11.3%	31 22%	44 31.3%	Total
	0.003							P-Value

## Discussion

The prevalence of neonatal seizures was 4.08% in this study while it is between 1.5 and 5.5 per 1,000 live births according to different studies (2-4). Female infants comprised the majority of patients in this study, whereas male infants comprised the majority of patients in the other studies (27-32) and males was a determined risk factor for neonatal seizures (32). In two studies (33, 34) it was shown that the majority of seizures occurred in neonates less than 10 days old. Calciolari (35) et al. indicated that 90% of seizures happened in infants at 2 days old. Dehdashtian et al. (30) showed that 70% of seizures occurred in the first 14 days of life. Mirzarahimi (31) indicated that seizures happened mostly in the second day of term infants and in first day of preterm infant. As we mentioned before, 78% of neonatal seizures happened in the first 10 days of life apparently because of delivery conditions and hypoxia. Therefore, we can reduce the incidence of neonatal seizure with improvement of delivery conditions.

In this study, 76% of patients had more than 2500 gr weight in compliance with Vanikim (36), which indicated that seizures could be more common in infants with heavier birth weights. In a similar study performed in Iran only, 22% of infants were less than 2500 gr weight (30) but seizures often happened within the aforementioned range of weight (19, 27, 33, 37). It can be justified that seizures occur in low-birth-weight infants because of underlying conditions such as sepsis, meningitis, and electrolyte abnormalities (30). In compliance with many other research and studies, hypoxemic-ischemic encephalopathy was the major reason of seizures in our study (2-4, 8, 30, 31, 35, 38, 39). In this study, metabolic disorders were considered as a second reason of seizure with 31 cases (22%), comprising 18 cases (58%) with hypoglycemia, and 13 (42%) cases with hypocalcaemia. Notwithstanding clinical, radiological, and laboratory studies, 36 cases (25.5%) had unknown reasons for seizures. In a similar study, 31% of patients had no significant reason for seizures (30) and Gabriel et al (40) classified 14% of seizures as unknown. Infectious causes (including sepsis and meningitis) in 16 cases (11.3%) were the third most common underlying factor in neonates with seizures. In another study (30) 6% of seizure causes were due to underlying infectious process. Brain hemorrhage (consisting of IVH and SAH) was another reason of seizure in patients. (30). The most common type of seizure was generalized and multifocal seizure with 70 cases (49.7%) while 56 infants (39.7%) suffered subtle seizures, and partial seizure happened in 15 infants (10.6%). Subtle and multifocal seizures were the most common seizures with 65% and 54%, respectively, in a study performed by Calciolari, et al (35). In another study, myoclonic (66%) and focal clonic (22%) were considered as the most common seizures (38). These findings comply with our results. 

There is a close relation between the causes of neonatal seizure and newborn death. In the present study 12.8% (18 cases) of the infants were expired, and among the causing factors, hypoxemic-ischemic encephalopathy by 33/3% (6 cases) was the factor with the highest mortality rate (P-value = 0.003). Moreover, there was a significant correlation between the neonatal mortality and the birth weight. Hypoxemic-ischemic encephalopathy was established as the leading cause of death in newborns suffering from neonatal seizures, which was consistent with previous studies in this respect (38, 41). Therefore, it is possible to reduce the mortality and morbidity rate associated with neonatal seizure by recognition of prenatal asphyxia risk factors and controlling these factors on one hand and early diagnosis and prompt and appropriate treatment on the other. 

Imaging studies such as CT scans and MRIs in patients affected by seizures can demonstrate a range of brain lesions that can lead to neonatal seizures. From another viewpoint, the sensitivity of the CT scan to show the brain lesions caused by hypoxic-ischemic encephalopathy are significantly higher than neonatal brain ultrasounds as indicated by radiology textbooks (42). MRIs are preferred over CT scans due to the non-ionizing radiation and the imaging method of choice in the neonatal period; however, it is relatively difficult to be performed on patients at this age (25). Further, in an emergency setting, CT scans are more practical (26). In two separate studies that evaluated seizures in neonates, performing a CT scan was indicated as an essential diagnostic measure (1, 43). Furthermore, based on two non-concurrent studies concerning prognosis of neonatal seizures, the extent of brain CT scan lesions were found to be an important factor in determining the prognosis of newborns affected by seizures. These studies emphasize the need to perform CT scans or MRIs in the evaluation of neonatal seizure (44, 45). Rennie et al. (46) ascertained the duration of treatment in newborns affected by seizures found that the extent of brain CT scan lesions was one of the determining factors. In Taghdiri research 65% of patients had abnormal CT scan results and brain hemorrhage in 23% neonates was the most common radiological finding (39). In this research, 33.3% patients had abnormal CT scan results and hypoxemic-ischemic encephalopathy in 47% neonates was the most common lesion observed in the brain CT. Presence of a notable abnormal brain CT scan in most patients complied with other references as well (18, 22). Accordingly, the occurrence of convulsive movements in the newborn can be an important indicator of a serious underlying lesion in the brain tissue; therefore, by using brain-imaging studies promptly, the cause of these movements can be established and following an appropriate treatment, the survival of the patient can be improved.

**In Conclusion, **a precise recognition of interfering factors in neonatal seizures are of prime importance in the guidance of a treatment plan and, thus, failure to identify these background factors leads to the impairment of neurological functions. Although there is significant controversy around the potential effects of seizures on the newborn’s brain, neonatal seizures background factors such as the underlying etiology and the extent of brain damage have unquestionable role in long-term consequences of the disease. 
